# Preference of *Polistes dominula* wasps for trumpet creepers when infected by *Xenos vesparum*: A novel example of co-evolved traits between host and parasite

**DOI:** 10.1371/journal.pone.0205201

**Published:** 2018-10-24

**Authors:** Laura Beani, Federico Cappa, Fabio Manfredini, Marco Zaccaroni

**Affiliations:** 1 Dipartimento di Biologia, Università di Firenze, Sesto Fiorentino (Firenze), Italia; 2 School of Biological Sciences, Royal Holloway University of London, Egham, United Kingdom; Indian Institute of Science, INDIA

## Abstract

The parasitic insect *Xenos vesparum* induces noticeable behavioral and physiological changes—e.g. castration—in its female host, the paper wasp *Polistes dominula*: parasitized putative workers avoid any colony task and desert the colony to survive in the nearby vegetation, like future queens and males do. In this long-term observational study, we describe the spectacular attraction of parasitized workers towards trumpet creeper bushes (*Campsis radicans*) in early-summer. Two thirds of all wasps that we sampled on these bushes were parasitized, whereas the parasite prevalence was much lower in our study area and most wasps sampled on other nearby flowering bushes were non-parasitized. First, we describe the occurrence and consistency of this phenomenon across different sites and years. Second, we evaluate the spatial behavior of parasitized wasps on *C*. *radicans* bushes, which includes site-fidelity, exploitation and defense of rich extra-floral nectaries on buds and calices. Third, we record two critical steps of the lifecycle of *X*. *vesparum* on *C*. *radicans*: the parasite’s mating and a summer release of parasitic larvae, that can infect larval stages of the host if transported to the host’s nest. In a nutshell, *C*. *radicans* bushes provide many benefits both to the parasite *X*. *vesparum* and to its host: they facilitate the parasite’s mating and bivoltine lifecycle, a phenomenon never described before for this parasite, while, at the same time, they provide the wasp host with shelter inside trumpet flowers and extrafloral gland secretions, thus likely enhancing host survival and making it a suitable vector for the infection.

## Introduction

Social insects are prime targets of parasites that can find suitable conditions within insect colonies to spread [1]. The primitively eusocial wasp *Polistes dominula* (Hymenoptera, Vespidae) is the host of the endoparasitic insect *Xenos vesparum* (Strepsiptera, Xenidae). This parasite is capable to induce significant modifications in the host physiology (including castration, [2–4]) and behavior. If parasitized by *X*. *vesparum*, *P*. *dominula* workers do not participate in colony tasks (e.g., they do not contribute to rearing siblings), do not receive aggressions by nest-mates and behave instead like future queens, i.e. they desert the nest to form summer aggregations on selected vegetation nearby [5–7]. This is a striking unusual behavior in a model organism for social evolution [8], and a recent study has shown a significant shift in the expression of caste-related genes that is associated with parasitism [9]. These off-season “estivation/hibernation gatherings”, first described by Hamilton [10], have been extensively documented in the past because they are aberrant and conspicuous, located for days on the same leaves, with a 98% parasite prevalence [5]. They usually occur in August and September in Tuscany (i.e. late in the wasp colony cycle) and include putative workers that have deserted the colony in June and July (hereafter defined as “parasitized workers”), and castrated putative queens that emerged and departed from their colony in August [11].

In this long-term observational study, we focused on the behavior of parasitized workers in early-summer (from the end of June until mid-July), immediately after their nest desertion and during the time-span preceding August aggregations. This set of field observations on a cryptic phase in the life of parasitized wasps complement a few laboratory reports previously published on this topic [5], as artificial rearing conditions may affect social dynamics in paper wasps [12]. We were able to carefully monitor the behavior of single parasitized workers, less conspicuous and observable than aggregations, thanks to their unexpected high prevalence on *Campsis radicans* bushes (Bignoniaceae), commonly known as “trumpet creeper”. This perennial plant, bearing long, tubular, orange flowers from June to September, is native to North America and naturalized in Europe, where it is widespread along fences, in wastelands and gardens [13]. This plant is regularly visited by several hymenopterans, due to its rich floral and extra-floral nectaries [14–16].

Here we describe for the first time the behavior of parasitized *P*. *dominula* workers on *C*. *radicans*. First, we quantify the rate of parasitized workers on *C*. *radicans* bushes in comparison to other flowering plants available in the same area, across seven years of collections and in different sites in Tuscany. Second, we compare site-fidelity towards trumpet creepers, and spatial and feeding behavior between parasitized and non-parasitized wasps. Third, we detect both the occurrence of mating and the summer release of triungulins of *X*. *vesparum* on *C*. *radicans* flowers. This is the first time that a bivoltine cycle–spring and summer release–for this parasitic insect is described. We hypothesize that the preference of parasitized wasps for trumpet creepers may be a case of interactive behavioral manipulation, whereby the parasite enhances the survival of the host (important vector of the infection and the winter reservoir of the parasite) by inducing the host to exploit the extra-floral resources of this plant. The altered spatial and feeding behaviors of parasitized wasps could be considered as host compensatory co-evolved traits, meeting the transmission objectives of the parasite [17].

## Materials and methods

### Ethics statements

The collection of wasps and the behavioral experiments comply with the current laws in Italy. No specific permits are required for collection of wasps and colonies. The species used in the experiments is not endangerd or protected in Italy.

### Parasite-host life cycle

**Fig 1:**
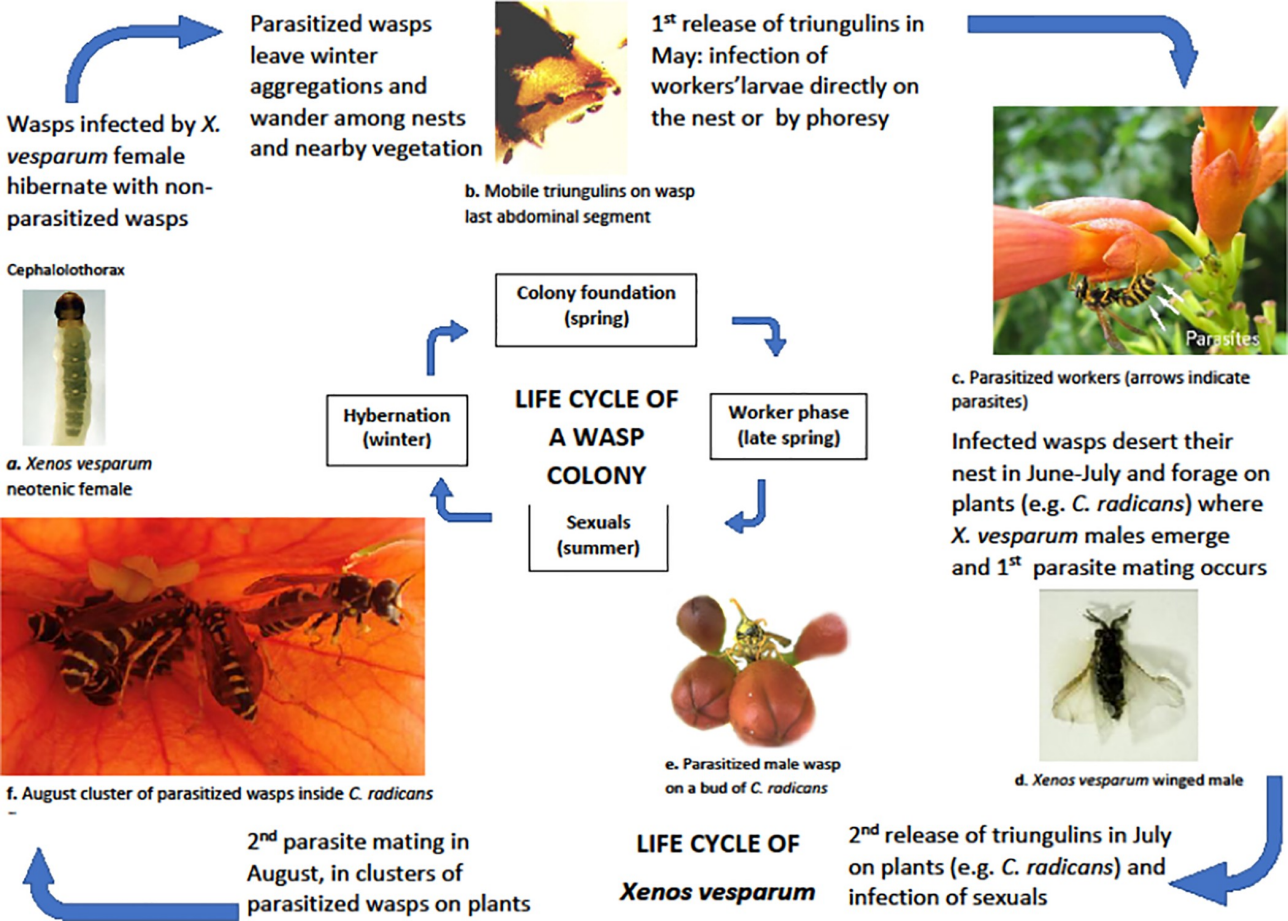
The life-cycle of the strepsipteran *Xenos vesparum* in parallel with the life-cycle of its primary host, the paper wasp *Polistes dominula*. **a)** *X*. *vesparum* female. An extragenital canal opens in the cephalothorax of the female, where both mating and larval escape occur. **b**) Triungulins escaping from female canal. **c)** Parasitized workers on *C*. *radicans*. Note the distortion of wasp abdomen due to parasites (arrows). **d**) *X*. *vesparum* male. Observe the striking sexual dimorphism of the parasite. **e)** A parasitized *P*. *dominula* male. **f**) Aggregation of parasitized wasps. Photos by Laura Beani.

In this system, parasite and host meet when they are both immature organisms (Fig 1). Tiny triungulins, the first infective stage of *X*. *vesparum*, enter into all larval stages of *P*. *dominula* [18, 19], while they are incapable to penetrate into adult wasps: hence the parasite cannot be transmitted horizontally among adults. The abdomen of a parasitized *P*. *dominula* wasp shows a typical irregular shape, due to the extrusion of the parasite between abdominal segments (see Fig 1). In summer, the winged male parasite emerges from his puparium, that protrudes from the host’s abdomen, and wanders in search of a receptive female to inseminate, dying shortly after mating [20]. The neotenic female parasite instead is permanently confined within the hemocoel of her host and may overwinter inside the hosts. In the spring, *P*. *dominula* wasps infected by *X*. *vesparum* females are the vector of the parasite transmission: mature triungulins leave their mother’s cephalothorax and are deposited directly on a wasp nest, where they can infect new larval hosts, or on flowers, where foraging non-parasitized wasps may accidentally pick them up and carry them via phoresy to their own nest [18, 21, 22].

### Sweep-netting

Our main sampling sites were four bushes in the plain of Sesto Fiorentino (Florence, Italy: 43° 50’ 7” N, 11° 11’ 46” E): two bushes of *C*. *radicans* (height: 2.5 m; length: 14 and 12 m, hereafter called A and B), one of *Rosa* L., cultivar (height: 2.5 m; length: 10, hereafter R) and one of *Jasminum officinale* L. (height: 2.5 m; length: 11, hereafter J). These 4 bushes, prominent landmarks in the flat landscape, all located within a range of 40 m from each other at the corners of a square fence, were intensively patrolled by *P*. *dominula* and other insects (*pers*.*obs*.). Wasps were collected across 7 years (2009–2015), froze and dissected to evaluate the proportion of parasitized and non-parasitized workers (*N* = 523, 15-min sampling per bush from 10.00 to 13.00). In July 2015 (1^st^ week of July, 15-min collection from 10.00 to 13.00), we collected, froze and dissected *P*. *dominula* wasps from 13 *C*. *radicans* bushes in 4 different sites in Tuscany where we previously found parasitized nests, to control if the preference for this plant was a local phenomenon. These bushes were prominent sunlit landmarks, similar in size to bushes A and B, far apart 10–40 m from each other. These locations are: Fornello (Pontassieve); Chiesina Uzzanese (Pistoia); Pieve a Nievole (Pistoia); Chiocchio (Greve in Chianti).

### Mark-recapture study

To evaluate site-fidelity to trumpet creepers, 658 *P*. *dominula* wasps (333 parasitized workers, 121 non-parasitized workers, 204 non-parasitized males) were collected from bush A, more attractive than bush B for wasps due to its rich blooming, marked on the thorax with fast-enamel paint and released near the same bush (2^nd^ and 3^rd^ weeks of July). At mid-July parasitized wasps were easily recognizable without dissection because the parasite was fully extruded from the wasp abdomen. This mark-recapture study was repeated across 3 years (2011–2013). We carried out 30-min daily censuses on bush A from 10.00 to 13.00 over 6 different days. The wasps that were recaptured at least three times after the first marking were considered as “resident”.

### Spatial and feeding behavior

Focal wasps were individually marked and video-recorded at 1 m distance, to avoid any interference (2^nd^ week of July 2015, 10-min scan periods, from 10.00 to 13.00, bush A). Wasps were collected, froze and dissected at the end of observations to verify parasite’s occurrence, ovary development and the amount of fat bodies. We selected 10 workers parasitized by one *X*. *vesparum* (6 with one male and 4 with one female) and 9 non-parasitized workers as control (small body size, no signs of wearing on wings, undeveloped ovaries, scant fat bodies). It was not possible to perform blind observations because the protrusion of the parasite from host’s abdomen is evident. We evaluated the frequency (N events/10 min) of the following behavioral categories: a) flight; b) bud patrol, i.e. feeding on buds and calices, touching the substrate with antennae and mandibles, further split into long bud patrol: 30–60 sec or more; and short bud patrol: < 30 sec; c) leaf patrol, i.e. inspection on leaves, < 20 sec; d) flower patrol, i.e. inspection of the outer corollas, < 20 sec; e) entry into flower; f) aggressive interaction with other wasps/insects approaching the focal wasp, usually chased away after a quick antennation.

### *X*. *vesparum* analyses: Mating trials, female dissections and larval control

Twelve wasps, collected on bush A and parasitized by one or two *X*. *vesparum* females, were individually enclosed into vials covered with a mesh large enough for a male parasite, but not for an infected wasp, to pass through, and hung on branches of bush A. After three hours we verified the occurrence of male parasites inside the vials as a proxy to quantify successful mating (1^st^ week of July 2010, from 10.00 to 13.00).

To assess the occurrence of mature triungulins in the brood canal of female parasites, we dissected under a stereomicroscope (Nikon 645 c-dss) *X*. *vesparum* females infecting two sets of workers. 1) Lab workers which emerged from nests collected in Sesto Fiorentino and were housed under controlled laboratory conditions (at 26 ± 2 C° and at a natural photoperiod, water and sugar cubes *ad libitum*). Workers harbouring parasites of both sexes were caged together until the end of July, when female parasites were analysed (2010, N = 30; 2011, N = 24). 2) Field parasitized workers (bushes A and B, mid-July, 2011–2014), from which 115 *X*. *vesparum* females were isolated and analysed. Moreover, we checked under a stereomicroscope the outer and inner corollas of 30 flowers for the presence of triungulins (bushes A and B, mid-July, 2011–2012).

### Statistical analysis

Chi-square test was carried out to compare: the proportion of parasitized and non-parasitized wasps across 7 years among 4 bushes; the site-fidelity among males, parasitized and non-parasitized females across 3 years. Seven spatial behaviors were used as dependent variables into Generalized Linear Model (GLM) to compare parasitized (10) and non-parasitized (9) wasps. We computed a matrix of distances among behavioral frequencies by means of Bray-Curtis dissimilarities, using the vegdist function of the Vegan package for R, and then a principal coordinates analysis (PCoA) with cmdscale R function. We extracted n-1 dimension. A scatterplot of the first two dimensions visualized the dissimilarity pattern of samples. The probability to attribute the sample to its group (parasitized/non-parasitized) was evaluated with a Jack-knife procedure. All the statistical analyses were conducted using SPSS 20.0 and R.

## Results

### Sweep-netting

**Fig 2:**
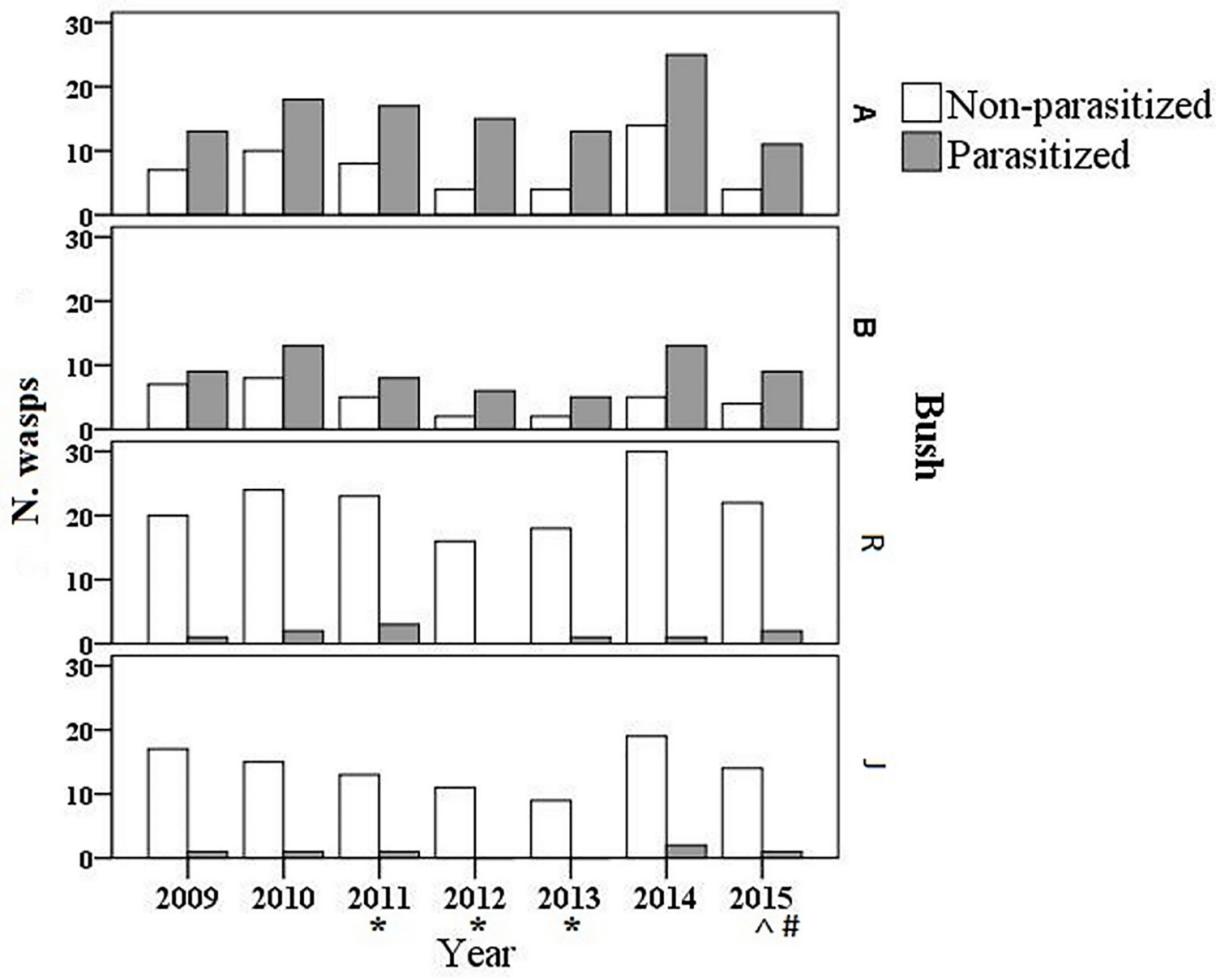
Sweep-netting collections of *P*. *dominula* wasps. Collections were carried out on two *C*. *radicans* bushes (A and B) and one bush each of rose and jasmine (R and J) across 7 years (2009–2015). By clumping together 7 years of collections, the percentage of parasitized wasps out of all wasps was 68.7% on A, 65.6% on B, 5.9% on R and 6.1% on J. Timeline of experiments: * years of Mark-recapture (2011–2013); ^ year of Focal observations (2015); # year of Sweep-netting outside Sesto Fiorentino (2015).

In this long-term field study (2009–2015) we sampled wasps from four flowering bushes, similar in size and sun exposure. Wasps were collected between late June and early July, i.e. during the peak of the worker phase in the colony life cycle. The number of *P*. *dominula* ranged between 10 and 30 at each sweep netting (Fig 2). Two thirds of *P*. *dominula* workers collected on trumpet creepers (bushes A and B) in June-July were parasitized (i.e. 175 parasitized and 84 non-parasitized wasps), while both rose and jasmine bushes (R and J) were visited mainly by non-parasitized wasps (95%, 248 non-parasitized and 16 parasitized wasps). This asymmetrical distribution of parasitized wasps, more numerous on trumpet creepers, was consistent with no significant difference across the 7 years of the study (chi-square-test, df = 6. A, χ^2^ = 2.33, P = 0.88; B, χ^2^ = 1.69, P = 0.94; R, χ^2^ = 1.85, P = 0.93; J, χ^2^ = 3.22, P = 0.78). The percentage of parasitized wasps collected on two trumpet creepers (A+B, clumped together because not significantly different from each other, χ^2^ = 0.26, P = 0.60) was 66–69%; this rate is significantly higher (χ^2^ = 213.35, P < 0.001) than the 6% of parasitized wasps collected on rose and jasmine bushes clumped together (not significantly different from each other, R+J, χ^2^ = 0.04, P = 0.94).

In August, during 3 years (2011–2013), sporadic checks on bushes A and B revealed the presence of *P*. *dominula* males and few aggregations of 2–7 parasitized wasps inside corollas, resting or seeking floral nectar (see August clusters, Fig 1**F**). We discovered 10 small clusters inside flowers of bush A (mean wasp number = 3.9) and 7 clusters on bush B (mean wasp number = 3.6). This late-summer phenomenon was never observed on rose and jasmine bushes.

To exclude that the attraction towards *C*. *radicans* was a local phenomenon, on July 2015 we sampled wasps from 13 flowering bushes of the same species far 30–40 km from our main sampling site. In these sweep-netting collections, parasitized wasps ranged between 22% and 77% (Table 1). A further mid-August screening showed that 8 bushes, first attractive for parasitized females, were then intensively patrolled by *P*. *dominula* males. Moreover, 7 small aggregations (mean wasp number = 2.9) were discovered inside the flowers of 3 bushes.

**Table 1:** Sweep-netting collections of *P*. *dominula* wasps from *C*. *radicans* bushes in 4 different locations in Tuscany (July 2015). Parasitized wasps were more abundant than non-parasitized wasps in 10 out of 13 bushes.

Location	Coordinates	Bushes	Wasps collected on each bush: N parasitized/N non-parasitized (% parasitized)			
			Bush 1	Bush 2	Bush 3	Bush 4
**Fornello**	43° 50’ N, 11° 50’ E	4	17/5 (77)	10/9 (57)	4/14 (22)	9/6 (60)
**Chiesina**	43° 50’ N, 10° 43’ E	4	18/8 (69)	11/5 (69)	10/5 (67)	4/9 (31)
**Pieve**	43° 52’ N, 10° 48’ E	3	17/7 (71)	10/8 (56)	3/9 (25)	
**Chiocchio**	43° 35’ N, 11° 19’ E	2	18/8 (69)	11/6 (65)		

### Mark-recapture study

**Fig 3:**
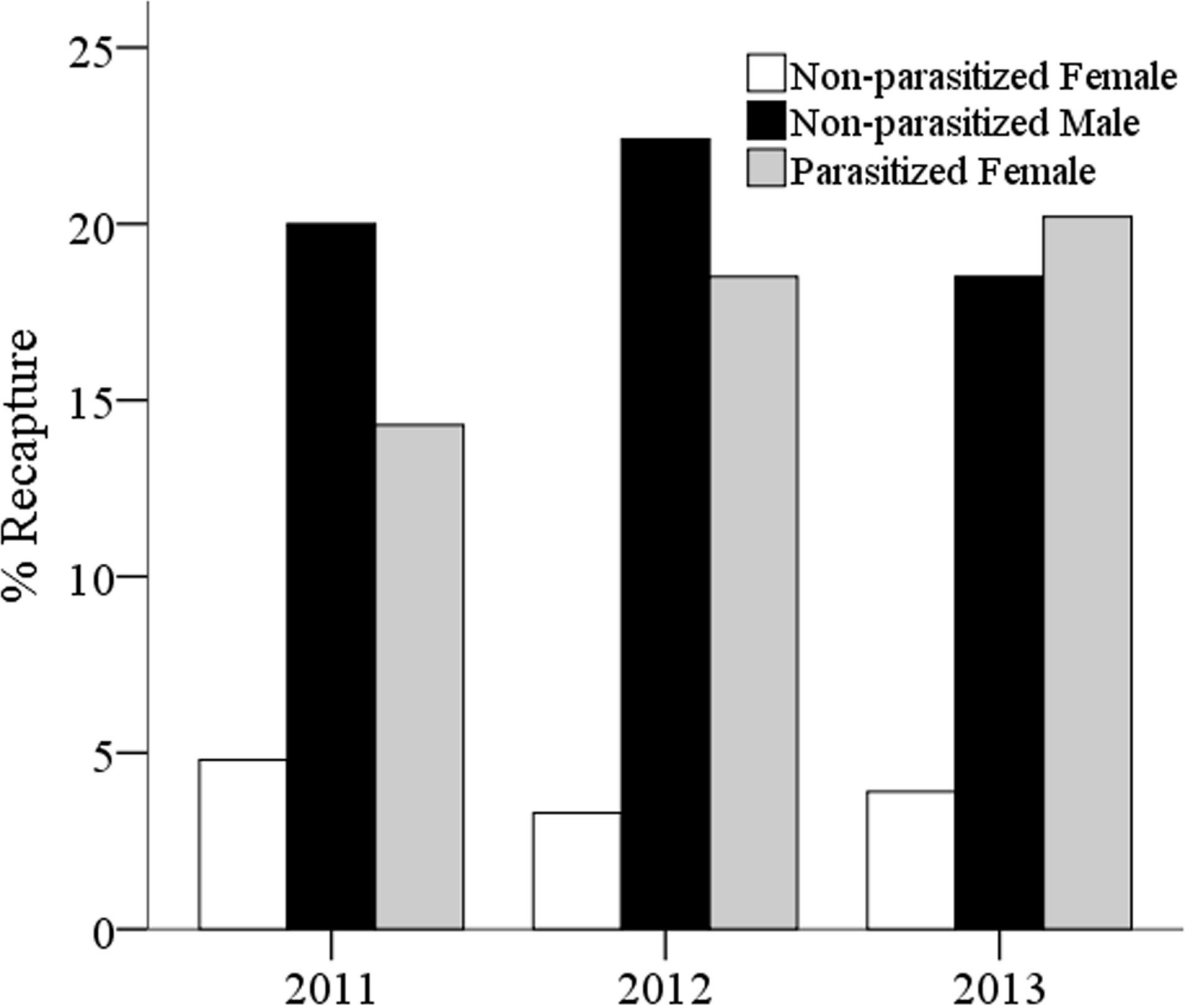
Mark-recapture of *P*. *dominula* wasps on bush A (2011–2013). Spatial and feeding behavior. Parasitized workers spent a long time feeding on inflorescences, touching the surface with antennae and mandibles. Their activity was focused mainly on buds and calices, which were carefully patrolled (Fig 4).

We evaluated site-fidelity (Fig 3) in relation to both wasp parasitism status and sex by means of a repeated mark-recapture study focused on bush A, the most attractive for parasitized wasps (see Fig 2). We recaptured a fifth of parasitized workers (N = 59 resident out of 333, 18%) on bush A for 3–5 days after their marking, and non-parasitized male wasps in a similar percentage (N = 45 resident out of 145, 22%; parasitized male wasps appear only later in the season because of the parasite bivoltine cycle, see below), while non-parasitized workers were observed much less frequently (N = 5 resident out of 121, 4% only, Fig 3). These rates were consistent across 3 years (chi-square-test, df = 2; Parasitized workers: χ^2^ = 1.04, P = 0.59; non-parasitized male wasps: χ^2^ = 0.08, P = 0.95; non-parasitized workers: χ^2^ = 0.08, P = 0.96 respectively) and significantly different between parasitized and non-parasitized workers (χ^2^ = 10.8, P = 0.001). Site-fidelity was not significantly different between parasitized wasps and males (χ^2^ = 1.02, P = 0.31).

**Fig 4:**
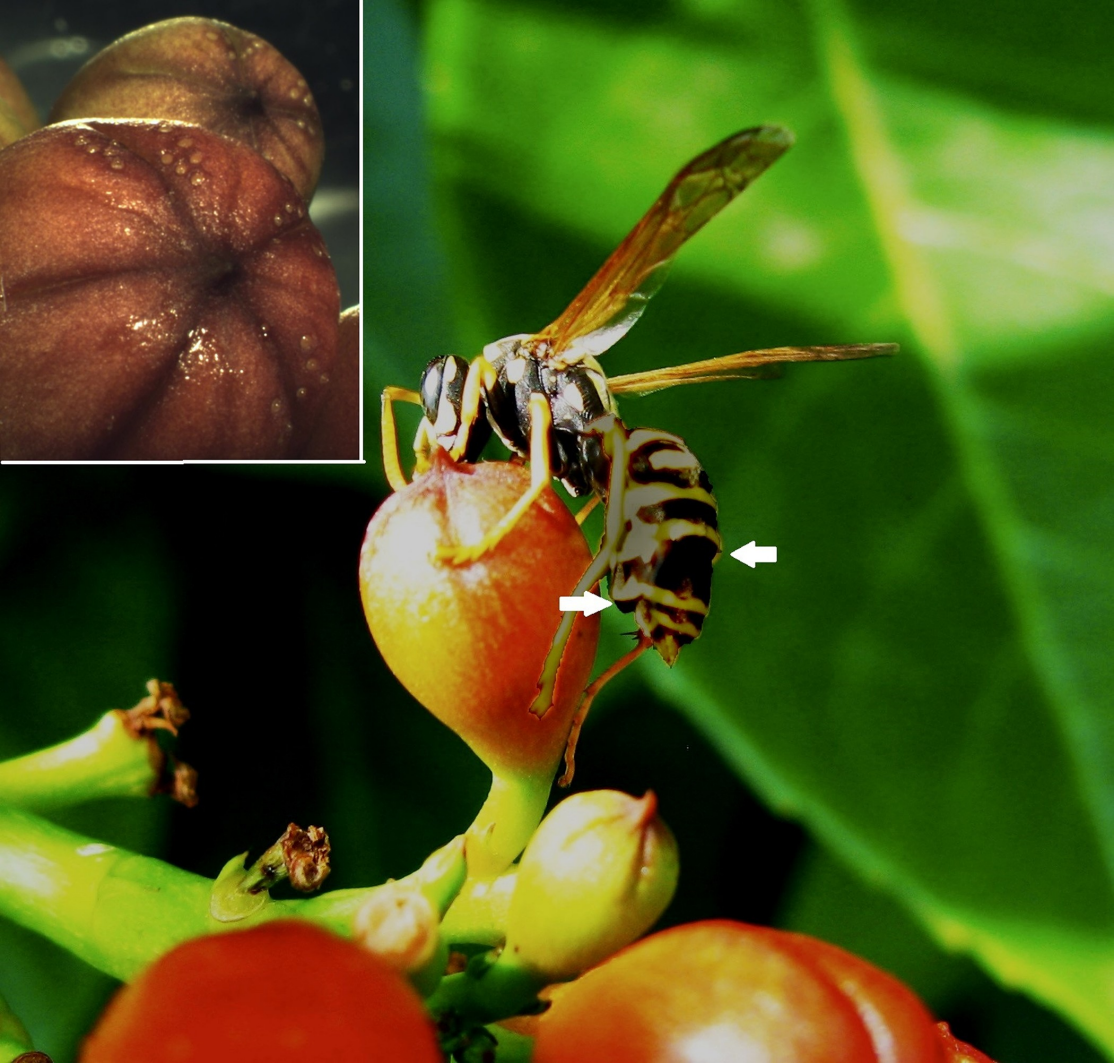
A parasitized wasp foraging on a closed bud of *C*. *radicans*. Note two puparia of *X*. *vesparum* extruding from the host abdomen, one left open after the male’s emergence and the other still closed (arrows). Photo by Laura Beani. In the inset, flower buds with several extra-floral nectaries. Photo by Corrado Tani.

**Fig 5:**
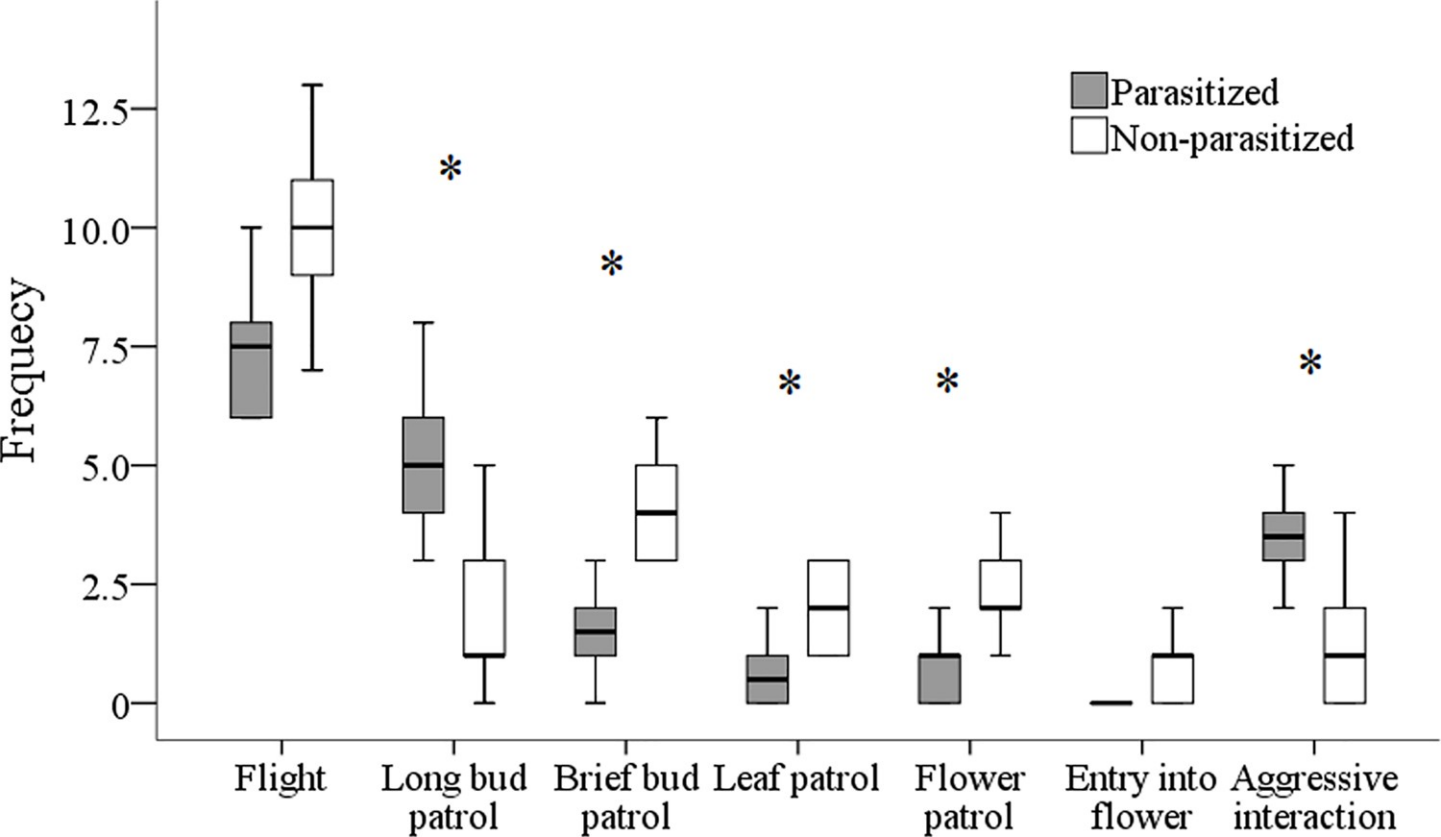
Behavioral frequencies of focal parasitized (N = 10) and un-parasitized (N = 9) wasps on bush A (events /10-min, July 2015). White box plots: un-parasitized wasps; grey box plots: parasitized wasps. Thick horizontal lines represent medians, boxes are upper and lower quartiles and whiskers indicate the highest and lowest values. (* P < 0.01).

Parasitized wasps spend more time (30–60 sec or more) than non-parasitized workers in long patrol of closed and open buds (Wald χ^2^ = 13.52, P < 0.01, Fig 5). Focal parasitized workers on buds chased out an approaching wasp or other insect more frequently than non-parasitized workers (aggressive interactions, Wald χ^2^ = 9.09, P < 0.01). Non-parasitized workers landed on these inflorescences briefly, flying quickly among buds, leaves and flowers; in comparison to parasitized wasps they showed short bud patrols (Wald χ^2^ = 9.08, P < 0.01), leaf and flower patrols (lasting 10–20 seconds, Wald χ^2^ = 6.91, P < 0.01 and Wald χ^2^ = 5.55, P < 0.01 respectively). Entry into flowers to search for ovarian nectary was rare and quick overall in comparison to what was observed for bees and other pollinators (*pers*. *obs*). Early in the morning, when wasps were inactive, we observed single wasps sheltering inside the tubular corollas, usually facing outwards, mainly during windy and rainy days. Three checks on 20 flowers, containing one wasp each, showed that the majority was parasitized (13, 17 and 18 parasitized out of 20 wasps, respectively; none was marked). No aggregations inside flowers were detected until August.

**Fig 6:**
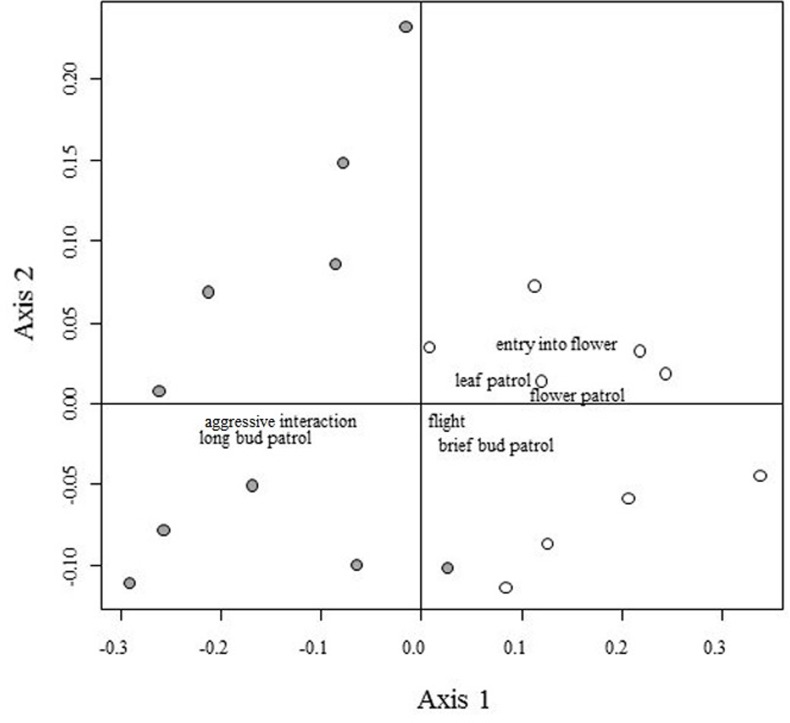
Scatterplot of the first two axes obtained by Principal Coordinates Analyses. The two groups of wasps (white circles = un-parasitized, grey circles = parasitized) are almost perfectly separated along the first axis.

The two-dimensional plot obtained after PCoA (Fig 6) showed a good separation between parasitized and non-parasitized groups on the first axis. The jack-knife procedure correctly attributed 17 on 19 specimens (89.5%). The high percentage of correct attribution of individuals to their groups supports the different behavioral frequencies between the two groups.

### *X*. *vesparum* analyses: mating vials, female dissection and larval control

Four out of 12 vials on bush A, enclosing wasps parasitized by *X*. *vesparum* females, harboured one dead *X*. *vesparum* male, while one vial contained two males, all dead at the time of our survey. Dissections of *X*. *vesparum* females, infecting lab workers which were housed under controlled laboratory conditions, showed groups of mature mobile triungulins inside the brood canal at the end of July, i.e. after 6–8 weeks from workers’ emergence (2010: 17 out of 30 *X*. *vesparum* females released triungulins, 57%; 2011: 24 out of 24 females, 100%). At mid-July, dissections of *X*. *vesparum* females infecting workers collected from bushes A and B confirmed the development of triungulins also in the field (2011: 32 out of 34, 94%; 2012: 26 out of 37, 70%; 2013: 2 out of 13, 15%; 2014: 13 out of 31, 42%). A screening of 30 *C*. *radicans* flowers collected from bushes A and B also showed a few mobile triungulins on the outer and inner corollas, 1–2 per flower (mid-July, 2011: 22 triungulins; 2012: 6 triungulins).

## Discussion

The current imbalance between empirical and theoretical studies on host manipulation by parasites [23] prompted us to describe the spatial and feeding behavior of *P*. *dominula* workers parasitized by *X*. *vesparum* in the field. These wasps are less conspicuous than wasps that form late-summer or winter aggregations. Nevertheless, we were able to describe their behavior in details thanks to the fortunate discovery of their consistent occurrence on *C*. *radicans* bushes over time and in different locations. Our observations show that parasitized *P*. *dominula* workers have a clear preference for *C*. *radican*s. Year after year, two-thirds of workers collected on these plants were parasitized, in comparison to only 6% as observed on other flowering bushes, which were intensively patrolled by non-parasitized wasps instead. This is an unexpectedly high rate, considering that the parasite prevalence in our study area is lower than in trumpet creepers (proportion of infected larvae and pupae per nest = 0.16 ± 0.7 [18]; proportion of infected adults in winter aggregations: between 0.10 and 0.25[24]). A recent screening on 14 parasitized nests, collected in Sesto area, confirms that 13.39% of workers were parasitized (30 out of 124 wasps emerged from these lab nests between June and July 2017, *pers*. *obs*.).

The results of this field study led us to refine our previous assumptions of parasitized wasps being “idle, gregarious zombies” [25]. This description still holds for parasitized wasps in late-summer, autumn and winter aggregations [5, 11, 26], while it is misleading for the parasitized workers of this study, that display a wide behavioral repertoire instead. If we consider our parasite-host system in the scenario of parasitic manipulation of host behavior [23], it comes natural to hypothesize that parasitized workers behave like active “puppets”, controlled by *X*. *vesparum* “puppeteers”, on selected bushes. First, the host, the infection vector, is capable to survive outside the nest because it finds shelter (inside flowers) and nutrients (mainly extra-floral nectar) on trumpet creepers. While other plants may also offer nutrients and shelter to parasitized wasps (*Morus*, *Vitis*, *Hedera*, *Cynara*, *Populus* spp., see [5]) trumpet creepers are unique because of their unusual richness in extra-floral nectaries [14, 15, 27]. Second, the high number of parasitized workers harbouring both female and male parasites likely facilitates encounters of male-female *X*. *vesparum* on trumpet creepers (as confirmed by our mating vials test). Third, the workers infected by fertilized *X*. *vesparum* females release triungulins on trumpet creepers and this creates an opportunity for the parasitic larvae to be transported to a new host nest via phoresy by non-parasitized wasps, foraging on these bushes.

The release of parasitic larvae has been usually reported in the spring [21, 24], when the targets of the infection are larvae of worker wasps that are the only available caste at that time of the year. The summer release of triungulins that we report here indicates that also larval stages of sexual wasps (males and putative queens) might be the targets of a later infection, and this supports the noticeable rate of parasitized males previously observed in the field (up to 30% in the Sesto Fiorentino area, Cappa et al., 2014). These are the first data about a bivoltine cycle of *X*. *vesparum*, and *C*. *radicans* bushes appear as a key element to mediate this complex process. Previously, Hamilton [10] wondered if “hyperactive” wasps “could spread triungulins fast enough in the summer to be a second generation of stylops in the nest” (the word stylops refers here to *X*. *vesparum* parasites: it first was adopted as a common name for the group). Hamilton suggested that early males could be the vectors of this phenomenon, while our observations indicate that the actors are the workers; nonetheless his hypothesis of a bivoltine cycle for *X*. *vesparum* is now supported.

The higher site-fidelity and the lower flight frequency of parasitized workers in comparison to foraging workers clearly denote the emergence of a new spatial behavior in the worker caste that could be induced by the parasite. This behavioral shift cannot be explained in terms of reduced energy that is available to parasitized workers, as the parasite stops absorbing nutrients from the host after extrusion [28], although mounting an immune response may be costly. Parasitized workers tend to persist on bushes: early in the morning they seek shelter inside flowers, whereas during the day they wander among inflorescences. The prolonged feeding on closed buds and calices of *C*. *radicans*, two structures with a high density of nectaries rich in sucrose and glucose [14–16], suggests that parasitized workers search for extra-floral nectar sources more intensively than non-parasitized workers. This is also supported by their defensive behavior towards *C*. *radicans* flowers and by their sporadic entry into flowers compared to non-parasitized workers.

*C*. *radicans* contains anti-oxidant and anti-inflammatory secondary metabolites (flavonoids, phenolic acids and coumarins) [29–31]. It will be interesting in the future to perform detailed chemical analyses of extra-floral nectaries and lab tests to measure wasp susceptibility to pathogens and/or survival after ingestion of this nectar. Previous studies have shown that parasitized wasps have high levels of expression of *Defensin* and other genes associated with immune functions [9, 32, 33]. This usually indicates that the immune system is activated; hence a selected feeding strategy of parasitized wasps on trumpet creepers could represent a complementary strategy to boost their immune-competence in response to parasitism. Gregarious but not social and deprived of any colony task, parasitized workers patrol selected plants, year after year, to survive outside the protective environment of the colony. As parasitized workers cannot achieve direct fitness (as they are sterile) nor indirect fitness (as they desert the colony), their wandering behavior on trumpet creepers only benefits the parasite fitness in the end, if *X*. *vesparum* enhances its transmission through mating and larval release on *C*. *radicans* flowers. Instead of supporting any exclusive categorization of host altered behaviors (e.g. non-adaptive infection by-product, facilitated parasite transmission or host adaptation reducing infective detriments), we are more inclined to think that the preference of parasitized wasps for trumpet creepers is a novel example of co-evolved traits between hosts and parasites (according to Lefèvre et al. [17]): a shared rather than an extended phenotype. In the hypothesized scenario, *X*. *vesparum* exploits the compensatory spatial and feeding behavior of *P*. *dominula* to allow the completion of its life cycle.

## Supporting information

S1 Table Wasp collections on 4 bushes (A, B, R, J) from 2009 to 2015 (see Fig 2).
(XLSX)

S2 Table Mark-recapture study on bush A from 2011 to 2013 (see Fig 3). (XLSX)

S3 Table Behavioral frequency/10 min of parasitized and non-parasitized wasps (see Fig 5). (XLSX)

S1 Fig One triungulin (length 300μm) on *C*. *radicans* flower under stereo microscope. (TIF)
