# Supplementary figures and images for: Preference of Polistes dominula wasps for trumpet creepers when infected by Xenos vesparum: A novel example of co-evolved traits between host and parasite
Source: PLoS One. 2018 Oct 24;13(10):e0205201. doi: 10.1371/journal.pone.0205201 (PMC6200222; doi:10.1371/journal.pone.0205201)

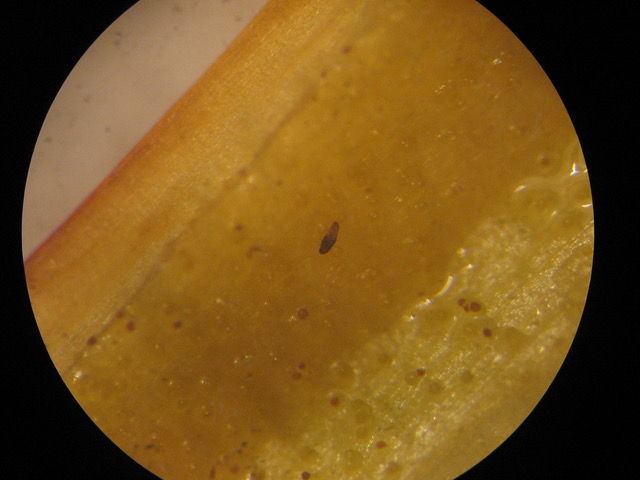

Supplement: S1 Fig — (TIF) [file pone.0205201.s004.tif]
